# Efficacy of lumbosacral and sacrococcygeal epidural ropivacaine in dogs undergoing surgery for perineal hernia

**DOI:** 10.3389/fvets.2023.1163025

**Published:** 2023-09-21

**Authors:** Kati Salla, Tuuli Åhlberg, Jaan Lepajoe, Ira Kallio-Kujala, Sari Mölsä, Daniela Casoni

**Affiliations:** Department of Equine and Small Animal Medicine, Faculty of Veterinary Medicine, University of Helsinki, Helsinki, Finland

**Keywords:** analgesia, dog, epidural, lumbosacral, sacrococcygeal

## Abstract

Epidural anesthesia is commonly administered as part of balanced anesthesia for perioperative analgesia. The main goal of this randomized clinical trial was to compare the efficacy of two epidural approaches in dogs undergoing surgery for a perineal hernia. A secondary aim was to compare motor blockade. Intact ASA 1 and 2 male dogs, weighing ≤25 kg with no previous surgery for perineal hernia were enrolled. After premedication with IM acepromazine 0.02 mg/kg and butorphanol 0.3 mg/kg, general anesthesia was induced with propofol and maintained with sevoflurane in oxygen. Dogs were randomly allocated to receive either a lumbosacral (LS, *n* = 30) or a sacrococcygeal (SC, *n* = 26) epidural injection with ropivacaine 1% (0.2 mL/kg) under computed tomography guidance. Successful analgesia was defined as no need of intraoperative rescue analgesia (fentanyl 3 μg/kg IV). Clinical failure was defined as the need of more than two boluses of fentanyl/h each dog received meloxicam 0.2 mg/kg IV at the end of the surgery. The Glasgow Composite Pain Scale short form (GCPS-SF), tactile sensitivity, pressure pain thresholds and motor blockade were assessed at 4, 6, 8, and 24 h after the epidural injection. Methadone (0.2 mg/kg, IV) was administered if the GCPS-SF was ≥6/24 points. Differences between groups were analyzed with the Mann–Whitney *U* test, Student’s *t*-test or Fisher’s Exact test, as appropriate. Success rate was assessed for non-inferiority between groups. The non-inferiority margin was set at −10%. Epidural analgesia was successful in 24 dogs in group LS and 17 dogs in group SC (*p* = 0.243), resulting in success rates of 80 and 65% in LS and SC groups, respectively. The non-inferiority of group SC versus group LS was confirmed. Clinical failure was recorded in two dogs in group LS and one dog in group SC. No significant differences between groups were detected in the GCPS-SF score, tactile sensitivity, pressure pain thresholds, need of post-operative methadone, or motor blockade. Both epidural techniques are valuable analgesic options for perineal hernia repair in dogs.

## 1. Introduction

Epidural analgesia is broadly considered to be the gold standard analgesic technique for major surgery, in both veterinary and human medicine. In human medicine, evidence suggest that, besides providing reliable pain relief, it obtunds the stress response to surgery and can reduce the incidence of postoperative pulmonary, thromboembolic and cardiac complications (1). In dogs, the use of epidural anesthesia has been shown to reduce the use of both intraoperative and postoperative rescue analgesia and to decrease plasma concentrations of stress response biomarkers during surgery (2–4). Epidural (or extradural) anesthesia entails the administration of local anesthetics into the epidural space, namely the potential space located between the *dura mater* and the wall of the vertebral canal. Both the epidural and the intervertebral space are typically large at the level of the lumbosacral junction, which is why this location is commonly selected for the epidural injection in small animals, as it gives the anesthetist the greatest chance of performing a successful block (5). The lumbosacral approach (LS; i.e., the puncture of the epidural space between the last lumbar and the first sacral vertebra) is a broad-spectrum technique of providing analgesia for surgery on the pelvis, pelvic limbs (2, 6) and the perineal and abdominal regions (7), as well as for operations on the thoracic area (8, 9). However, the LS approach in dogs carries a risk of inadvertent intrathecal injection. The occurrence is reported not only in small-breed dogs, in which the *conus medullaris* extends to the sacral region (10, 11), but also in large-breed dogs, despite the expected extension of the *conus medullaris* only as far as the sixth or seventh lumbar vertebra (12). Among the unwanted effects of epidural anesthesia cardiovascular depression and long-lasting motor blockade of the pelvic limbs are often reported. Cardiovascular depression is the result of preganglionic sympathetic blockade directly related to the cephalad spread of the block. Motor blockade of the pelvic limbs is a result of a non-selective blockade of L4–S1 spinal nerves (5). Motor blockade is usually self-limiting over time but may not be desired during recovery. Therefore, there is potential for the use and investigation of more caudal approaches, particularly when a cranial migration of the anesthetic is neither necessary nor desired. A sacrococcygeal approach (SC; i.e., the puncture of the epidural space between the last sacral and the first coccygeal vertebra or between the first and the second coccygeal vertebra) is routinely used in large animals to provide analgesia of the perianal region, but the evidence regarding dogs and cats is scarce. In particular, the technique has been used with success for catheterization and pain management in the treatment of feline urethral obstruction (13). More recently, the technical aspects of a sacrococcygeal injection have been investigated in a comparative study on dogs (14), but no clinical comparison between the intra- and postoperative effects of an LS versus an SC epidural injection has been carried out in dogs.

The primary aim of the current study was to compare the efficacy of the two epidural approaches in dogs undergoing surgery for a perineal hernia in terms of successful intraoperative antinociception and freedom from postoperative analgesia. The secondary aim was to compare the safety of the two epidural approaches as regards intra-operative cardiovascular depression and postoperative motor blockade. We hypothesized that the efficacy of SC epidural anesthesia would be non-inferior to LS epidural anesthesia and that the former would cause less cardiovascular depression and motor blockade.

## 2. Materials and methods

This study was approved by the Animal Experiment Board of Finland (ESAVI/4467/04.10.07/2017) and performed with informed owner consent.

According to the sample size calculation^1^, a minimum of 56 epidural injections were needed to be 80% sure that the lower limit of a one-sided 95% confidence interval was above the non-inferiority limit of −10%. The margin of the non-inferiority limit was based on a percentage difference in the epidural efficacy representative of a significant clinical difference, with the estimated success rates of 80 and 60% for LS and SC, respectively.

The inclusion criteria were ASA I–II, male dogs, weight less than 25 kg, a uni- or bilateral perineal hernia with no previous surgical corrections and normal locomotor activity. The exclusion criteria were ASA III or higher, weight > 25 kg, age > 12 years, skin infection at the site of the epidural injection or the inability to blind the responsible investigator to the epidural injection.

Dogs were randomly allocated to receive either an LS or SC epidural injection, and two-block randomization was used according to the surgical technique^2^. The perineal hernia was corrected by using either the elevation of the internal obturator muscle (EIOM) technique (15) or the autologous fascia lata graft (FLG) technique (16). In the case of a bilateral perineal hernia, only one side was operated on, and the contralateral side was corrected 2–4 weeks later with the same surgical technique. Dogs that underwent two operations were anesthetised with both epidural techniques. Each dog was castrated with the prescrotal technique during the first surgical session.

On the morning of the surgery, a clinical examination was performed and baseline measurements of tactile sensitivity and pressure algometry were obtained from the same two points in the perineal area, i.e., approximately 3 cm from the dorsal and ventral edge of the estimated line of incision. For tactile sensitivity, von Frey filaments (Aesthesiometer II, Somedic SenseLab, Hörby, Sweden) ranging from 0.0064 to 24 g of nominal force (corresponding 3.906–93.023 g/mm^2^) were used. After the von Frey filament testing, pressure pain thresholds were measured with a mechanical pressure algometer (FDN 100–Algometer, Wagner Instruments, USA) with a blunt-tipped area of 1 cm^2^. The algometer was regularly calibrated according to the manufacturer. The pressure was increased at a rate of approximately 2 N/s from 10 N (lower detection limit) until a behavioral response was elicited from the animal or the applied force reached 30 N (cut-off). A threshold of 8 N was attributed when the dog showed a behavioral response at contact with the probe.

The dogs were premedicated with IM 0.02 mg kg^−1^ acepromazine (Plegicil 10 mg/mL, Bela-Pharm GmbH, Germany) and 0.3 mg kg^−1^ butorphanol (Butordol, 10 mg mL^−1^, Intervet Internarional B.V., Netherlands). Anesthesia was induced 30 min later with IV propofol (Propovet Multidose 10 mg mL^−1^, Zoetis Animal Health, Copenhagen, Denmark) at a dose of 2–4 mg kg^−1^ to allow tracheal intubation. Anesthesia was maintained with sevoflurane in oxygen, targeting an absent palpebral reflex, with eyes rotated and loose jaw tone. Heart rate (HR) and rhythm, non-invasive blood pressures, oxygen saturation of hemoglobin, as well as pulse rate, respiratory rate (RR), end-tidal carbon dioxide and osophageal thermometry were monitored in each dog. Active forced-air warming was provided throughout the procedure, with the aim of maintaining a core temperature of above 36°C.

Before the epidural injection, hair was clipped to allow both epidural techniques. After positioning the dogs in sternal recumbency, ropivacaine 1% (0.2 mL kg^−1^) was administered into either the LS or the SC epidural space under imaging guidance using a helical 64-slice multidetector computed tomography (CT) scanner (Lightspeed VCT, GE Healthcare, Madison, WI, USA). Correct positioning of the epidural needle (Spinocan, 22G, B. Braun Medical Industries, Germany) between the vertebrae was verified with CT, and the penetration of the epidural space was verified with the hanging drop and the loss of resistance method (5). After the injection, the clipped area was covered with adhesive tape to ensure blinding to the epidural technique.

During the surgery, a fixed end-tidal sevoflurane concentration of 2.3% was targeted. Epidural analgesia was defined as successful when no intraoperative rescue analgesia was needed and as partially successful if a maximum of two boluses of fentanyl were required. Intra-operative fentanyl (Fentanyl Hameln, 50 μg mL^−1^; Hameln Pharma Plus Gmbh, Germany) at a dose of 3 μg kg^−1^ IV was administered if two out of three parameters (HR, RR and mean arterial pressure [MAP]) were increased by more than 20% from the baseline values recorded before the surgical incision. Clinical failure was declared if more than two boluses of fentanyl *per* hour were required. In such cases, a fentanyl constant-rate infusion was initiated at a rate of 3–10 μgkg^−1^ h^−1^ and continued until the end of the procedure.

Hypotension was defined as a MAP of less than 60 mmHg, and bradycardia was defined as a HR of less than 60 bpm. Hypotension was treated according to clinical recommendations (17) – i.e., a crystalloid bolus, followed by dopamine constant-rate infusion at a rate of 2.5–10 μg kg^−1^ min^−1^. Bradycardia with concomitant hypotension was corrected with glycopyrrolate 0.01 mg kg^−1^ IV. Cardiovascular depression was recorded if interventions to stabilize cardiovascular function were initiated.

Before prescrotal castration, lidocaine (4 mg kg^−1^; Lidocain, 20 mg mL^−1^; Orion Pharma, Finland) was injected intratesticularly. Each dog received meloxicam 0.2 mg kg^−1^ IV (Metacam, 5 mg mL^−1^; Vetcare Ltd., Finland) at the end of the procedure. The duration of the procedure and the time between extubation and the first assessments were recorded.

The Glasgow Composite Pain Scale short form (GCPS) (18) scoring and the motor blockade of the pelvic limbs according to Tarlov’s score [(19); Supplementary File 1] were assessed at four, six, eight and 24 h after the epidural injection. Methadone (0.2 mg kg^−1^ IV; Insistor 10 mg mL^−1^; Richter Pharma, Austria) was administered if the GCPS score was ≥6/24, or ≥ 5/20 in non-ambulatory dogs. In addition, von Frey filament sensitivity and pressure pain thresholds were measured at the same time points after the epidural injection. All pain assessments and sensitivity threshold measurements were performed by the same experienced investigator (KS), who was blinded to the epidural technique.

Dogs were kept in the hospital overnight, and standard care with regular pain assessments was carried out every 4 h by a senior student or a nurse. Methadone was administered as described above, but these assessments were not included in the statistical analyses.

### 2.1. Statistical analyses

The intraoperative HR and MAP recorded before and 30 min after the epidural injections, as well as before and at 30, 60, and 90 min after the surgical incision for herniorrhaphy, were used for statistical analyses. Moreover, the need for intraoperative fentanyl or postoperative methadone, or the cardiovascular depression and the initiation of treatment for it before the epidural injection, were recorded as yes or no. In the case of clinical failure, the postoperative recordings for these dogs were omitted from the statistical analyses. If methadone was administered as rescue analgesia postoperatively, the following GCPS-SF, algometer and von Frey recordings were excluded from the analysis. Regarding the von Frey and pressure algometry measurements, the most sensitive recordings from the side operated on at a predetermined time point was used for statistical analyses and compared between the epidural techniques. Prior to statistical analyses, the von Frey filaments were coded (Supplementary File 2). Data were analyzed with SPSS software, version 27 (IBM SPSS Statistics, IBM Corp., Armonk, NY, USA). The normality of data distribution was evaluated with the Shapiro–Wilk test. Repeatedly measured continuous cardiovascular data were analyzed by means of mixed-model analysis of variance with post-hoc Bonferroni adjustment at selected time points. Parametric data measured only once were compared using unpaired two-tailed *t*-tests. Categorical variables and non-normally distributed variables were compared between the groups with the Mann–Whitney *U* test. For categorical variables recorded as yes/no, Fisher’s Exact test was applied. Non-inferiority of SC to LS was claimed if the lower limit of the 95% of confidence interval (CI) for the difference in success rate was greater than −10%. This test for non-inferiority was only performed for the primary outcome variable (success rate) if superiority was not demonstrated between the groups; all other variables were tested for the superiority of LS versus SC. Data are presented as mean ± standard deviation (SD) or median (minimum–maximum), as appropriate. An alpha level below 0.05 was considered statistically significant.

## 3. Results

Data were collected between June 2017 and December 2020.

**Figure 1:**
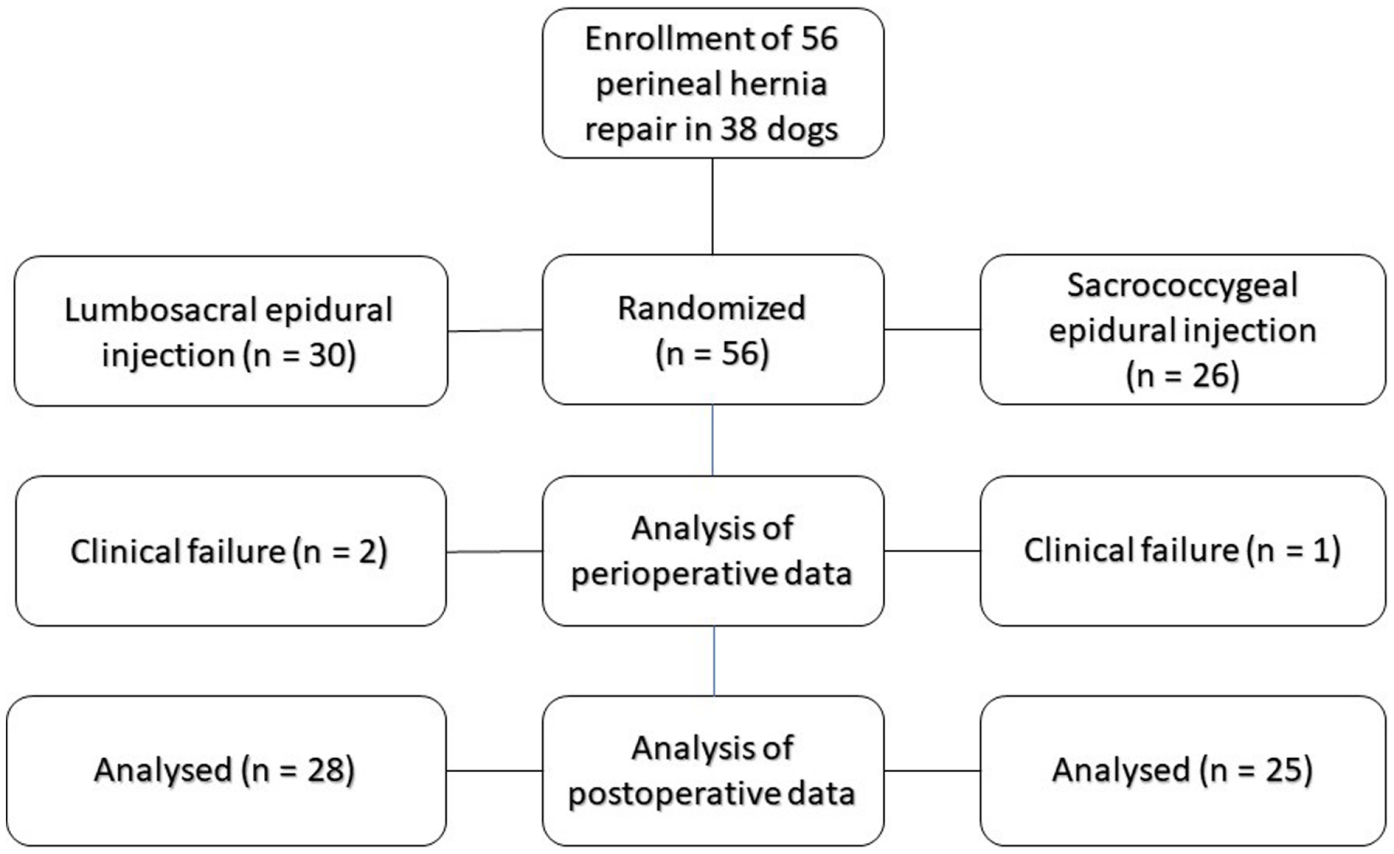
Consort diagram. Flow diagram of the “Efficacy of lumbosacral and sacrococcygeal epidural ropivacaine in dogs undergoing surgery for perineal hernia” -study according to CONsolidated Standards Of Reporting Trials (CONSORT).

A total of 56 perineal hernia repairs with epidural injections were carried out in 38 male dogs (Figure 1). Of these dogs, 18 (10 in group LS and 8 in group SC) underwent a bilateral operation with a period of 22 ± 9 and 30 ± 16 days between the procedures in groups LS and SC, respectively (*p* = 0.227). In total, the numbers of LS and SC epidural injections were 30 and 26, respectively. The respective weights of the dogs in groups LS and SC were 12.8 ± 6.6 kg and 14.1 ± 6.5 kg (*p* = 0.434). The ages of the dogs were 8.0 ± 1.64 years in group LS and 8.0 ± 1.63 years in group SC (*p* = 0.876).

A perineal hernia was repaired 33 times with the EIOM technique and 23 times with the FLG technique. As regards the epidural techniques, EIOM was used 19 times in group LS and 14 times in group SC, whereas FLG was used 11 times in group LS and 12 times in group SC. There were no differences in surgical techniques between the two epidural techniques (*p* = 0.588). The total duration of surgery, including prescrotal castration, was 114.5 ± 33.3 min in group LS and 135.0 ± 37.2 min in group SC (*p* = 0.034); when divided by surgical technique, the duration of the procedure was 111.5 ± 35.6 min for the EIOM technique and 141.96 ± 29.8 min for the FLG technique (*p* = 0.001). Twenty-six perineal hernia repairs were carried out on the left and 30 on the right side.

In all, 24 dogs in group LS and 17 dogs in group SC did not need rescue fentanyl during the procedure (*p* = 0.243), resulting in success rates of 80 and 65% in the LS and SC groups, respectively. The lower limit of the 95% CI of the difference for success rate (−8.2, 38%) was greater than the margin set for non-inferiority (−10%), therefore confirming the non-inferiority of the SC versus the LS technique. Partial success was achieved in 4 dogs in group LS (13%) and in 8 dogs in group SC (31%; *p* = 0.190). Clinical failure was recorded for two dogs in the LS group and for one dog in the SC group.

The time between extubation and the first assessments of GCPS-SF, Tarlov’s score, tactile sensitivity and algometry was 1.4 ± 0.7 h in group LS and 1.3 ± 0.6 h in group SC (*p* = 0.528). In addition to the recorded clinical failures, two dogs did not take part in the post-operative assessments due to their temperament being unsuitable for hospitalization. In all, methadone was administered post-operatively to 17 out of 51 dogs; out of these 17, 10 belonged to the LS group (10 out of 27; 37%) and 7 to the SC group (7 out of 24; 29%) (*p* = 0.767). In the 17 dogs receiving methadone post-operatively, the median (minimum–maximum) time of administration was 8 (6–24) and 7 (4–24) hours after the epidural injection in groups LS and SC, respectively (*p* = 0.449).

The results regarding post-operative pain assessments and motor blockade are presented in Table 1. There were no significant differences between the groups in GPCS-SF scores, pressure algometry, tactile sensitivity or Tarlov’s score at any of the time points. In addition, the percentage of dogs with a certain Tarlov’s score is presented in Table 2.

**Table 1:** The results of the short form Glasgow Composite Pain Scale (GCPS-SF), pressure algometer recordings, tactile sensitivity measurements according to categorized reactions to the von Frey filaments, and Tarlov’s score assessing motor blockade at baseline before the surgery (BL) and at 4, 6, 8 and 24 h after lumbosacral (LS, *n* = 27) or sacrococcygeal (SC, *n* = 24) epidural injections with 1% ropivacaine (0.2 mL kg^−1^) in dogs undergoing perineal hernia repair.

		BL	4 h	6 h	8 h	24 h
GCPS-SF	LS	na	1 (1–3)	2 (1–5)	3 (1–5)	2 (1–7)
	SC	na	2 (1–4)	2 (1–3)	4 (2–4)	2 (0–6)
*p*-values		na	0.113	0.873	0.283	0.562
Algometer (*N*)	LS	22.6 ± 7.9	25.5 ± 9.6	21.6 ± 9.0	16.9 ± 9.1	17.9 ± 9.1
	SC	22.2 ± 7.0	26.3 ± 9.3	22.7 ± 10	23.4 ± 9.7	19.2 ± 9.8
*p*-values		0.925	0.838	0.796	0.138	0.762
vonFrey	LS	0 (0–4)	0 (0–3)	0 (0–5)	3 (0–5)	2 (0–5)
	SC	0 (0–4)	0 (0–4)	1 (0–5)	1 (0–5)	0 (0–4)
*p*-values		0.415	0.276	0.401	0.942	0.417
Tarlov’s score	LS	>4	0 (0–4)	3 (1–4)	4 (3–4)	4 (4–4)
	SC	>4	3 (0–4)	4 (0–4)	4 (3–4)	4 (4–4)
*p*-values		na	0.343	0.619	0.168	1.000

Data are presented as mean ± standard deviation or median (minimum–maximum). *P* < 0.05 was considered statistically significant. na, not assessed.

**Figure 2:**
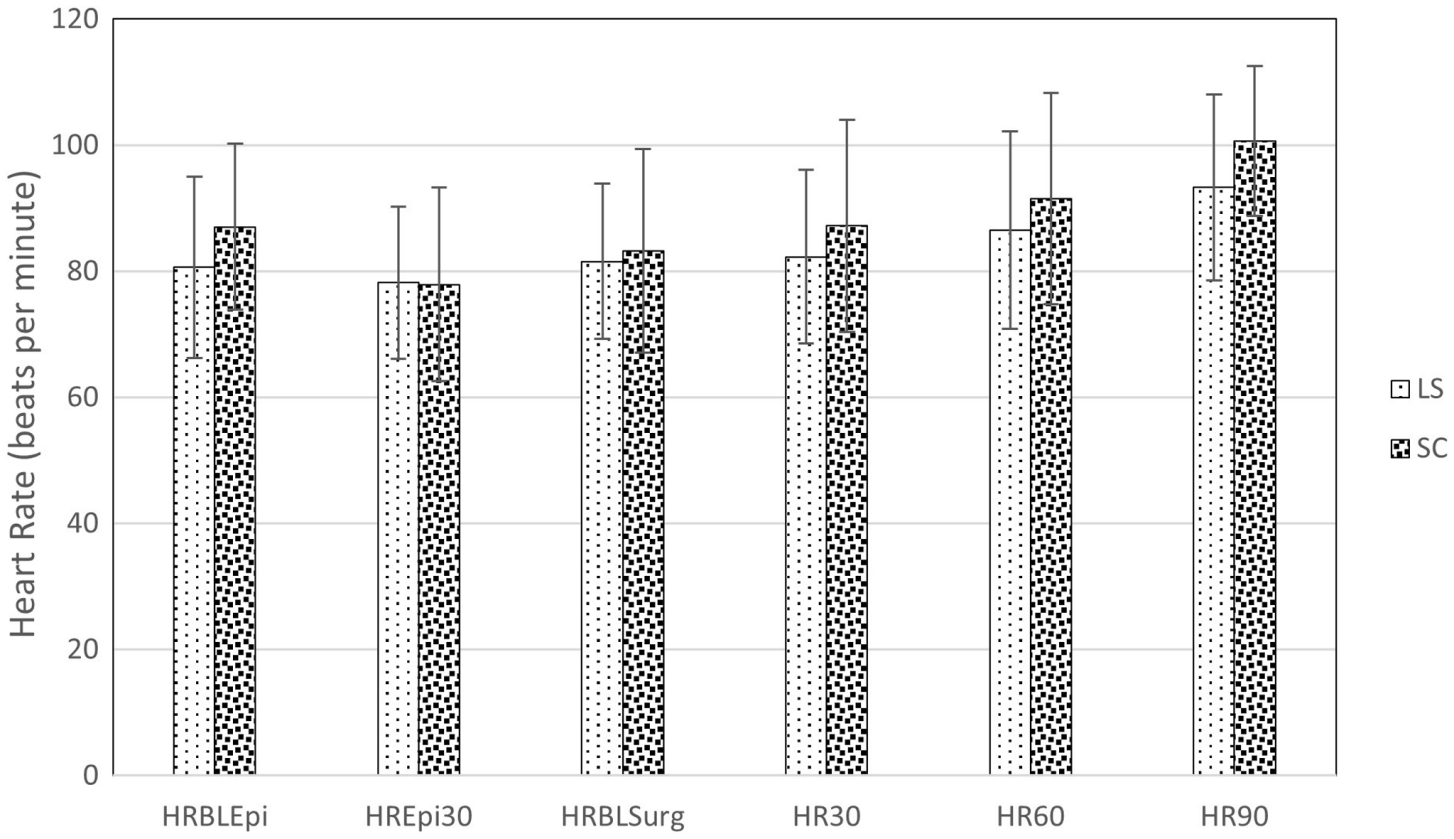
Heart rate (HR) in dogs at the following time points: before (BLEpi); 30 min after (T30Epi) lumbosacral (LS, *n* = 30) or sacrococcygeal (SC, *n* = 26) epidural injections with 1% ropivacaine (0.2 mL kg^−1^); baseline before perineal hernia repair (BLSurg) and 30 (T30Surg), 60 (T60Surg) and 90 (T90Surg) minutes from the incision. Data are presented as mean ± standard deviation. No significant difference (*p* < 0.05) between LS and SC group was detected.

**Figure 3:**
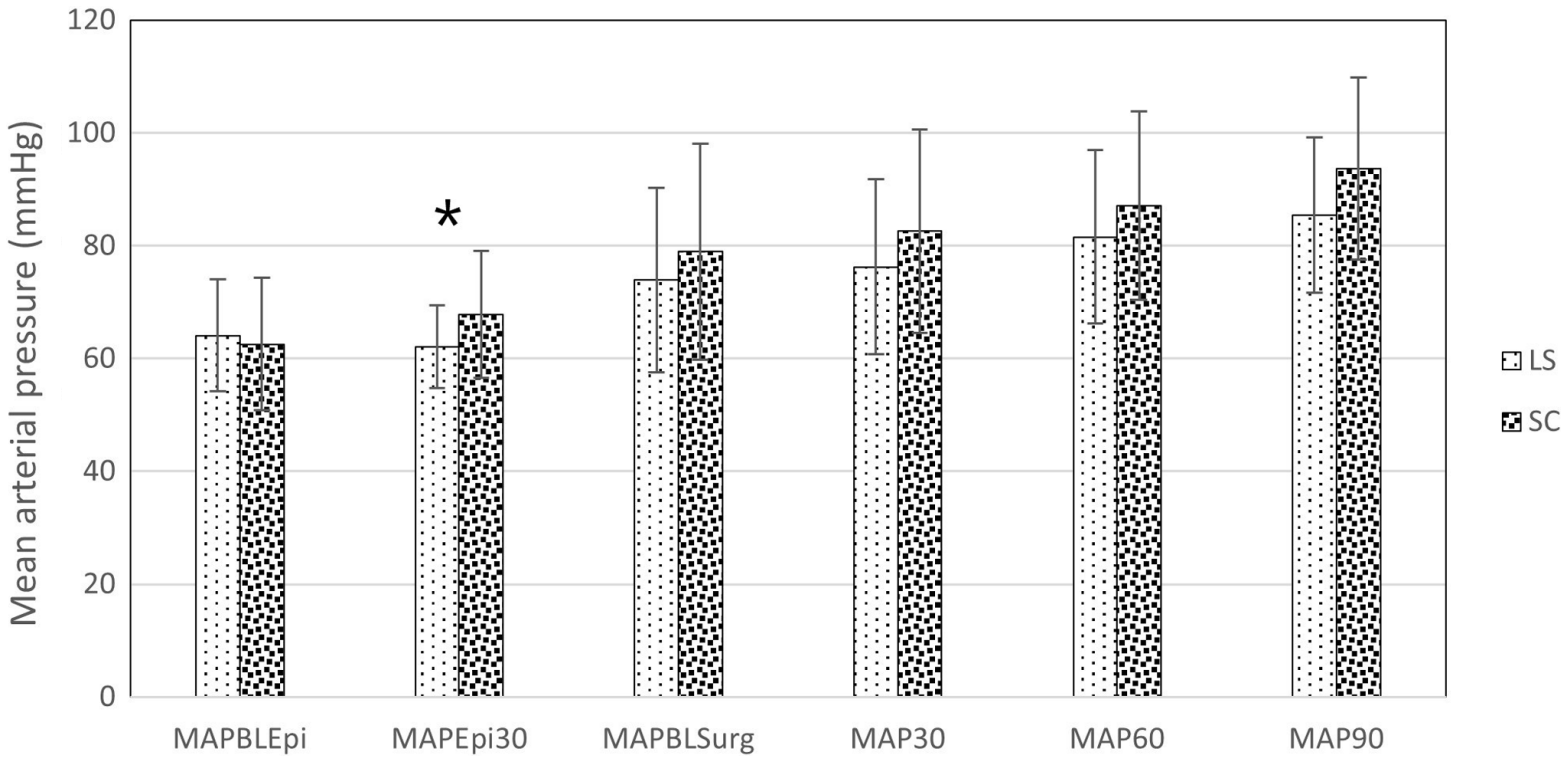
Mean arterial pressure (MAP) in dogs at the following time points: before (BLEpi); 30 min after (T30Epi) lumbosacral (LS, *n* = 30) or sacrococcygeal (SC, *n* = 26) epidural injections with 1% ropivacaine (0.2 mL kg^−1^); baseline before perineal hernia repair (BLSurg) and at 30 (T30Surg), 60 (T60Surg) and 90 (T90Surg) minutes from the incision. Data are presented as mean ± standard deviation. *Significant difference between LS and SC groups (*p* < 0.05).

Cardiovascular data from the selected time points are presented in Figures 2, 3. At 30 min after the epidural injection, MAP was significantly higher in group SC than in group LS (*p* = 0.027). Moreover, HR decreased significantly in both groups (*p* = 0.034) after the epidural injection, but no significant difference between the groups was detected (*p* = 0.364). The HR (*p* = 0.524) and MAP (*p* = 0.795) recorded during the perineal hernia surgery did not differ significantly from the baseline values recorded before the incision. In addition, there was no significant difference between group LS and group SC in either HR (*p* = 0.086) or MAP (*p* = 0.09) over time during the perineal hernia procedure.

**Table 2:** The percentages (%) of dogs with a certain Tarlov’s score (0–4) assessing motor blockade at 4, 6, 8 and 24 h after lumbosacral (LS, *n* = 27) or sacrococcygeal (SC, *n* = 24) epidural injections with 1% ropivacaine (0.2 mL kg^−1^) in dogs undergoing perineal hernia repair.

Tarlov’s score	Epidural technique	Timepoint			
		4 h	6 h	8 h	24 h
0 (%)	LS	55	0	0	0
	SC	40	10	0	0
1 (%)	LS	22	23	0	0
	SC	14	5	0	0
2 (%)	LS	0	23	0	0
	SC	0	20	0	0
3 (%)	LS	6	12	36	0
	SC	33	25	17	0
4 (%)	LS	17	38	64	100
	SC	13	40	83	100

In total, cardiovascular depression was recorded in 46 dogs; in 28 of these, the cardiovascular depression and its treatment started before the epidural injection. Eighteen dogs showed cardiovascular depression after the epidural injection, and 10 of these dogs belonged to group LS and 8 to group SC (*p* = 1.000). None of the dogs needed further treatment after the surgery.

## 4. Discussion

The main finding of this clinical study was that the SC epidural technique was efficacious and non-inferior to the LS epidural technique when the epidural success rate was compared in dogs undergoing perineal hernia surgery with or without castration. Interestingly, the success rate of the SC technique was even higher than expected (65% versus the expected 60%). Moreover, only 37% of the dogs in the LS and 29% dogs in the SC group needed rescue opioids in the postoperative period, with no difference between the epidural techniques. Cardiovascular depression requiring treatment was observed in several dogs before the epidural injection (28). The dogs in which the epidural injection triggered cardiovascular depression were fewer (18), with no difference between the groups. Although we did not verify the cephalad spreading of the anesthetic in our study, no differences were previously found in greyhound cadavers when the same volume that was used in our study (0.2 mL kg^−1^) was compared (20). Several factors have been described to influence the cephalad spread of an epidural anesthetic in living dogs, such as the size and weight of the dog as well as the length of the spinal canal, the size of the intervertebral foramina, the amount of fat in the epidural space and the direction of the needle (21–23). In order to limit confounders, we included dogs which were not heavier than 25 kg, and we always directed the bevel of the needle cranially; however, not all of the factors influencing the spread were controllable, which mirrors the biological variety of the canine species.

In our study, we did not aim to demonstrate the analgesic efficacy of epidural anesthesia for perineal hernia repair *per se,* and, therefore, a group which did not receive an epidural injection was not included. Some individuals needed a single bolus of fentanyl during the surgical procedure. The result is not completely surprising, as 1 mg kg^−1^ ropivacaine, when injected as the sole analgesic in the epidural space, has been previously reported to be insufficient in avoiding intraoperative nociception in dogs (6). The reasons for this could include an unequal distribution of ropivacaine over paravertebral nerves, distal roots in the subarachnoid space, and a spinal cord that is responsible for mediating the analgesic effect of epidural anesthesia (11).

Duration of sensory and motor block after 0.22 mL kg^−1^ of 0.75% ropivacaine administered epidurally was reported to last 133 ± 32 min (24). In our study, a multimodal analgesic regimen was applied for ethical reasons, including, in addition to epidural ropivacaine, routine intraoperative intratesticular lidocaine and a single dose of postoperative meloxicam in both group LS and group SC. However, intratesticular lidocaine for prescrotal castration could be redundant in case of clinical success of epidural as the anatomical extent of the block should desensitize the nerves of testicular plexus, the visceral afferent fibers of which derive from the fourth, fifth, and sixth lumbar ganglia (25). When administered as part of balanced anesthesia, epidural ropivacaine resulted in a longer postoperative analgesic effect than previously reported (26). The duration of epidural blockade has been reported to have linear relationship to the concentration and dosage of bupivacaine (27). The same might applies to ropivacaine, and in general to local anesthetics. Indeed Campoy et al. (28), suggested that once the nerves are blocked, a greater concentration of local anesthetic serves to increase the intensity and the duration of effect.

Interestingly, there were individuals in both groups that did not need any rescue analgesia during the postoperative phase, based on the repeated assessment with GCPS. Therefore, using either of these epidural techniques during perineal hernia repair in combination with a postoperative non-steroidal analgesic should be considered an option for an opioid-sparing analgesia protocol. Additionally, the results of our study highlight the importance of tailoring pain management based on pain assessments. This finding is in line with the results of the study by Bini et al. (29), who demonstrated that the consumption of postoperative rescue opioid analgesics in dogs decreased when the analgesics were administered according to repeated pain assessments instead of fixed intervals.

Combining the GPCS-SF with pressure algometer and tactile sensitivity assessment supports our findings regarding the protracted analgesic effects during the postoperative phase in this population of dogs. Although these dogs had an existing pathology and could therefore not be considered naïve to pain before surgery, no signs of allodynia in the peri-incisional region were detected after the application of either epidural technique.

We did experience some short-term motor blockades in both group and we were not able to detect superiority of the SC technique in this by using Tarlov’s score. However, it should be noted that, independently of the epidural technique, each dog included in the study was able to walk without assistance within 8 h of the epidural injection, underlying the fact that the motor blockade induced by ropivacaine, when used at this dose and this concentration, was not long-lasting. It might well be that using lower volume of injectate, could have brought about a lower incidence of the motor blockade in both groups (30). However, the consequent reduction of dose of local anesthetic might have corresponded to a lower intensity and duration of sensory block as well. It should also be noted that Tarlov’s score lacks a scoring for normal locomotor activity (number 5) and we could therefore not determine whether there was a difference in the speed of returning to normal locomotor activity between the two groups. Interestingly, although this finding was not statistically significant, the median score in group SC at 4 h after the epidural injection was already 3, indicating that the dogs were able to bear their weight, whereas in group LS the median score at the same time point was 0, indicating no movement of the pelvic limbs. More frequent assessments or combining with other methods for assessing motor blockade could have been useful in detecting minor differences between the groups. In both groups, once able to walk, all the dogs were also able to urinate. However, in our study we did not specifically record the time to first urination, therefore any further comparison would be inappropriate.

Due to the clinical nature of the study, the duration of surgical procedures was not standardized and the surgical technique was not taken into account in the randomization. Indeed, FLG was used more often in group SC than in group LS, causing longer procedure times in group SC, although this difference was, in our opinion, not clinically relevant. The duration of the procedure and, therefore, the perpetuation of a nociceptive stimulus was longer in group SC, and this could be considered a finding that supports the non-inferiority of the SC versus LS technique. In addition, this result can be transposed to other clinical scenarios where the duration of the procedure is not fixed.

The use of epidurally administered local anesthetic agents for analgesia in sedated dogs has been reported to cause less cardiovascular depression in comparison to general anesthesia (30). On the other hand, epidural bupivacaine has also been reported to decrease mean arterial pressure in anesthetised dogs (31, 32). In our study, cardiovascular depression, according to the defined criteria, was detected in several individuals during general anesthesia, but it mostly occurred before the epidural injection. The severity of cardiovascular depression seems, according to some recent reports, to be influenced by the volume and concentration of the local anesthetic. Indeed, when ropivacaine was injected into the thoracic epidural space, the MAP decreased significantly in humans only at a dose of ropivacaine 0.75% but not with ropivacaine 0.375% or 0.2% (33). In dogs undergoing a cesarean section, the use of lumbosacral epidural analgesia (ropivacaine, bupivacaine or lidocaine) at a maximum volume of 0.3 mL kg^−1^ did not exacerbate hypotension (34). In our study, MAP differed between the SC and LS group at 30 min after the injection, but since the MAP was above the set threshold for treatment in both groups, this information might be not clinically relevant. Furthermore, we did not verify the spreading of the analgesic, nor was the type of recumbency a controlled variable at this time point, and this result should, therefore, be interpreted with caution. In addition, considering the limitations of the oscillometric technique, the finding should be confirmed with an invasive blood pressure technique.

The correct intervertebral space was verified with diagnostic imaging. The insertion of the needle into the epidural space was guided with the hanging drop and the loss and lack of resistance technique (35). However, actual proof of the needle tip being in the epidural space would have required epidurography with an injection of a contrast medium. As this was a clinical trial, this approach was not implemented to avoid potential adverse effects.

## 5. Conclusion

Both epidural techniques were valuable options in the context of multimodal balanced anesthesia for perineal surgery in dogs. Neither epidural technique was accompanied by clinically significant complications.

## Data availability statement

The raw data supporting the conclusions of this article will be made available by the authors, without undue reservation.

## Ethics statement

The animal studies were approved by Animal Experiment Board of Finland. The studies were conducted in accordance with the local legislation and institutional requirements. Written informed consent was obtained from the owners for the participation of their animals in this study.

## Author contributions

KS: study design, data collection, analysis and interpretation of data, and writing the original draft of the manuscript. TÅ, JL, IK-K, and SM: study design and data collection. DS: study design, data collection, analysis, and interpretation of data. All authors contributed to review and editing of the manuscript.

## Conflict of interest

The authors declare that the research was conducted in the absence of any commercial or financial relationships that could be construed as a potential conflict of interest.

## Publisher’s note

All claims expressed in this article are solely those of the authors and do not necessarily represent those of their affiliated organizations, or those of the publisher, the editors and the reviewers. Any product that may be evaluated in this article, or claim that may be made by its manufacturer, is not guaranteed or endorsed by the publisher.

## Footnotes

^1^ http://powerandsamplesize.com/Calculators/Compare-2-Proportions/2-Sample-Non-Inferiority-or-Superiority

^2^ www.randomization.com
