# Supplementary material for: Efficacy of lumbosacral and sacrococcygeal epidural ropivacaine in dogs undergoing surgery for perineal hernia
Source: Front Vet Sci. 2023 Sep 21;10:1163025. doi: 10.3389/fvets.2023.1163025 (PMC10551457; doi:10.3389/fvets.2023.1163025)
Supplement: Supplementary file 1 [file Data_Sheet_1.PDF]

Supplementary file 1. Tarlov's Score (Adami et al., 2016)

Grade	Description
0	Flaccid paraplegia, no movements of the pelvic limbs, possible loss of bolwel/urinary bladder control
1	Spastic paraplegia with moderate or vigorous purposeless movements of the pelcvic limbs. No sitting, unable to walk
2	Good movements of the pelvic limbs but unable to stand
3	Able to stand but unable to walk normally, hips and pelvic limbs obviously unstable, moderate to severe ataxia
4	Able to stand and walk normally, some muscle weakness of the pelvic limbs may be seen
