# Supplementary material for: Efficacy of lumbosacral and sacrococcygeal epidural ropivacaine in dogs undergoing surgery for perineal hernia
Source: Front Vet Sci. 2023 Sep 21;10:1163025. doi: 10.3389/fvets.2023.1163025 (PMC10551457; doi:10.3389/fvets.2023.1163025)
Supplement: Supplementary file 2 [file Data_Sheet_2.PDF]

Supplementary file 2. Categorization of the reactions to the von Frey filaments.

Colour	Diameter (mm)	Nominal Force (g)	g/mm^2	Points
Transparent	0.128	0.064	3.906	5
Brown/Orange	0.153/0.177	0.14 / 0.32	5.983 / 10.214	4
Blue	0.306	1.7	18.162	3
Black/Green	0.403/0.409	5.1 / 8.3	31.404 / 49.463	2
Yellow	0.508	24	93.023	1
No reaction	No reaction	No reaction	No reaction	0
